# On the potential role of intestinal microbial community in hepatocarcinogenesis in chronic hepatitis B

**DOI:** 10.1002/cam4.1550

**Published:** 2018-05-15

**Authors:** Ashraf Mohamadkhani

**Affiliations:** ^1^ Liver and Pancreatobiliary Disease Research Center Digestive Disease Research institute Shariati Hospital Tehran University of Medical Science Tehran Iran

**Keywords:** *Escherichia coli*, HBV‐carrier patients, hepatocellular carcinoma, intestinal microbiota, lipopolysaccharide

## Abstract

The chronic infection of hepatitis B virus (HBV) is the most potent risk factor for the development of cirrhosis and hepatocellular carcinoma (HCC). The association of intestinal microbiota alteration with progressive liver disease has been investigated in recent studies. Overgrowth of potentially pathogenic bacteria of gram‐negative species and, in particular, a significant increase in the fecal count of *Escherichia coli* (*E. coli*) are characterized in the presence of HCC. This study was conducted to describe the characteristics of the intestinal microbiota related to the presence of HCC in HBV‐carrier patients. The available literature indicates the colonization of *E. coli* as principal source of portal vein lipopolysaccharide (LPS), in the gut may contribute to the carcinogenesis process by inducing chronic inflammation. This understanding could help to predict the clinical outcomes in HBV‐carrier patients and innovative strategies to reduce the virulence of liver disease from intestinal dysbiosis.

## 1 INTRODUCTION

The intestinal microbiota in healthy adults creates a complex ecological system that plays an important role in the digestion of a wide variety of foods, in the synthesis of vitamins and in the breakdown of toxins and drugs.^1^ In addition, the microbial components of the intestine, which includes tryptophan catabolites, short‐chain fatty acids and bile acids, act as functional signaling molecules for well‐balanced microbial effects in the liver‐intestine axis. The normal composition of the intestinal flora, the maturation of the immune system, and the intestinal mucosal barrier organize the healthy intestinal functional barrier which breaks down into microbial dysbiosis.^2^ Chronic hepatitis with either hepatitis B or hepatitis C viruses (HBV or HCV) infections is associated with a greater translocation of the intestinal microbiota.^3^, ^4^ Intestinal microbiota alteration appears to have a significant role in the progression of hepatocarcinogenesis in HBV‐carrier patients.^5^, ^6^ This review summarizes the composition of the intestinal microbiota in chronic hepatitis B (CHB). We have also referred to the mechanisms that *Escherichia coli* (*E. coli*) may imply in hepatocarcinogenesis.

## 2 CURRENT KNOWLEDGE OF THE INTESTINAL MICROBIOTA

The intestinal microbiota is the most heterogeneous group of microorganisms that is rich in species, but unique as a fingerprint for each person. Half of the stools are virtually microbial biomass with about 10^13‐14^ microorganisms that are packed so densely with an extraordinary 10^11 ^cells/cm.^7^ The intestinal microbiota includes about 100 times more genes than the number of human genes. This characterizes human as meta‐organisms and the intestinal microbiota as our second genome.^8^, ^9^ Microbial diversity in the human gastrointestinal tract represents a challenge in characterizing microbial biomass as they are unidentified by current culture methods.^10^ However, the use of modern culture‐independent techniques based on variations in 16S rRNA genes revealed the presence of different species of more than 70 genera in the human gut.^11^ The taxonomic classification of the intestinal microbiota according to the ordinary nomenclature (phylum‐class‐order‐family‐genus‐species) has characterized the most common phyla *Firmicutes*,* Bacteroidetes*,* Actinobacteria* and *Proteobacteria*. *Firmicutes*, the main group, includes gram‐positive anaerobic bacteria with a low G + C content in the gastrointestinal tract. The *Bacteroidetes* are rod‐shaped anaerobic bacteria characterized in gram‐negative and nonspore. *Actinobacteria* such as gram‐positive and *Proteobacteria* as gram‐negative bacteria are also dominated in the gut. Despite the relatively stable intestinal microbiota, dysbiosis occurs in immune reactions, in inappropriate environmental and nutritional circumstances that are associated with a number of pathological situations.^9^, ^12^, ^13^

## 3 INTESTINAL MICROBIOTA IN HBV‐CARRIER LIVER DISEASE

Recent study in the diversity and composition of the intestinal microbiota in HBV‐induced chronic liver disease revealed alterations in the colonization of opportunistic intestinal bacteria. The intestinal microbiota of CHB patients has significant difference compared to healthy controls with increased bacterial charge in taxonomy: *Actinomyces*,* Clostridium sensu stricto*, unclassified *Lachnospiraceae* and *Megamonas* that influence on disease progression.^14^ Complicated patients with liver cirrhosis and liver cancer demonstrate intestinal dysbiosis that is also approved in animal studies.^4^, ^15^ In general, the composition of intestinal microbiota in HBV‐carrier patients shows an increase in the growth rate of *E. coli*.^15^, ^16^, ^17^ The intestinal microbiota profile of end‐stage liver disease in CHB patients showed an obvious increase in *Enterobacteriaceae*,* Enterococcus faecalis* and *Faecalibacterium prausnitzii*.^1^, ^6^ Intestinal bacteria with high frequency in CHB patients are illustrated in Table 1. Reciprocally, the lower number of intestinal lactic acid species such as *Lactobacillus*,* Pediococcus*,* Weissella* and *Leuconostoc* has been reported in CHB patients.^1^, ^6^ Furthermore, the amount of *Clostridium spp* cluster *XI* and *XIVa* as well as *Bacteroides*,* Prevotella,* and *Lactobacillus spp*. such as *L. rhamnosus* and *L. fermentus* was also adjusted with a concomitant decrease in fecal samples of HBV‐related cirrhosis.^6^, ^18^ The *Bifidobacteria*/*Enterobacteriaceae* (B/E) ratio also decreased significantly.^6^ Table 2 presented bacteria with lower frequency in HBV‐carrier patients. Cirrhotic patients with abundant intestinal *E. coli* are more susceptible to the development of HCC.^17^ Similarly, patients with liver cirrhosis and HCC showed higher levels of endotoxin in serum.^16^, ^19^ Antibiotic treatments (eg Norfloxacin) and the use of probiotics improve intestinal microbiota in CHB patients by increasing the number of *Bifidobacterium* and *Lactobacillus* with moderation in the amount of *Enterobacter spp*. that result in reducing the levels of systemic.^20^, ^21^

**Table 1:** Bacteria of common inhabitant of the human gastrointestinal system that increased HBV‐carrier patients

Bacteria	Bacteria classification	Characteristics	Reference
*Enterobacteriaceae*	Proteobacteria; Gammaproteobacteria; Enterobacteriales; *Enterobacteriaceae*	Gram‐negative bacteria including harmless symbionts and pathogens. LPS is produced by the death of bacteria elicit strong.	6, 31, 37
*Escherichia coli (E. coli)*	Proteobacteria; Gammaproteobacteria; Enterobacteriales; *Enterobacteriaceae*;* Escherichia*;* E. coli*	Gram‐negative, facultatively anaerobic, rod‐shaped, common in the lower intestine, mostly are harmless. Higher frequency of intestinal *E. coli* associated with the progression of liver disease	6, 41
*Enterococcus faecalis*	Eubacteria; Firmicutes; Bacilli; Lactobacillales; Enterococcaceae; Enterococcus; *E. faecalis*	Gram‐positive, commensal bacterium inhabiting the gastrointestinal tracts classified as part of the group D Streptococcus	6
*Faecalibacterium prausnitzii*	Firmicutes; Clostridia; Clostridiales; Clostridiaceae; Faecalibacterium; *F. prausnitzii*	Commensal bacteria of the human gut microbiota producing butyrate and other SCFAs	6
*Clostridium sensu stricto*	Firmicutes; Clostridia; Clostridiales; Peptostreptococcaceae	Clostridium cluster I species (Clostridium *sensu stricto*)	
*Clostridium difficileClostridium perfringens*	Firmicutes; Clostridia; Clostridiales; Clostridiaceae; *Clostridium*	Gram‐positive bacteria with capability of producing endospores, an important cause of diarrhea	37
*Veillonella*	Firmicutes; Negativicutes; Vellionellales; Veillonellaceae; *Veillonella*	Gram‐negative anaerobic cocci. well known for its lactate fermenting abilities	37

**Table 2:** Bacteria of common inhabitant of the human gastrointestinal system with lower frequency in HBV‐carrier patients

Bacteria	Bacteria classification	Characteristics	Reference
*Lactobacillus*	Firmicutes; Bacilli; Lactobacillales; Lactobacillaceae; *Lactobacillus*	Rod‐shaped, gram‐positive, nonspore‐forming bacteria of the family Lactobacillaceae. with ability to produce lactic acid	6, 18
*Pediococcus*	Firmicutes; Bacilli; Lactobacillales; Lactobacillaceae; *Pediococcus*	Gram‐positive lactic acid bacteria, within the family of Lactobacillaceae, solely homofermentative.	6
*Weissella*	Firmicutes; Bacilli; Lactobacillales; Leuconostocaceae; *Weissella*	Gram‐positive bacteria, within the family Leuconostocaceae, with varied morphology from spherical or lenticular cells to irregular rods that previously grouped along with Lactobacillus *spp*., Leuconostoc *spp*., and Pediococcus *spp*	1, 6
*Leuconostoc*	Firmicutes; Bacilli; Lactobacillales; Leuconostocaceae; *Leuconostoc*	Gram‐positive bacteria, within the family of Leuconostocaceae, along with other lactic acid bacteria are responsible for the fermentation of cabbage.	1, 6
*Clostridium* spp clusters XI and XIVa	Firmicutes; Clostridia; Clostridiales; Peptostreptococcaceae	16S rRNA gene sequences divided clostridial species into 19 clusters. The Peptostreptococcaceae are a family of Gram‐positive bacteria in the class Clostridia.	6, 18, 37
*Bacteroides*	Bacteroidetes; Bacteroidia; Bacteroidales; Bacteroidaceae; *Bacteroides*	Gram‐negative, obligate anaerobic bacteria, the most substantial portion of the mammalian gastrointestinal flora in whom eat plenty of protein and animal fats, benefit their host by eliminating potential pathogens from gut colonization	6, 37
*Prevotella spp*	Bacteroidetes; Bacteroidetes; Bacteroidales; Prevotellaceae; *Prevotella*	Gram‐negative bacteria, predominantly for those who eat more carbohydrates	6
*Bifidobacteria*	Actinobacteria; Actinobacteria; Actinobacteridae; Bifidobacteriales; Bifidobacteriaceae; Bifidobacterium	Gram‐positive, ubiquitous inhabitants of the gastrointestinal tract. Some bifidobacteria are used as probiotics. HBV‐carrier patients decreased the Bifidobacteria/*Enterobacteriaceae* ratio	6

## 4 THE IMPORTANCE OF MICROBIAL BYPRODUCTS IN ASSOCIATION WITH CHRONIC HEPATITIS B

The advantage of intestinal bacteria under normal physiological condition in the synthesis of essential products such as short‐chain fatty acids (SCFA) and secondary bile acids in metabolic homeostasis is well known. The produced SCFAs by commensal and probiotic bacteria carry out the transcriptional pathways through epigenomic modifications.^14^, ^22^ The blood circulation of intestine with microbiota‐derived nutrients and metabolites is directed to the liver via the hepatic portal vein and the liver, in turn, has a reactive impact on intestinal functions beyond the biliary secretion in the intestinal lumen.^22^ Bacteria are mainly active in the proximal colon, where the availability of the substrate is in the maximum capacity. In the proximal colon, the nondigestible carbohydrates are hydrolyzed by the bacteria into oligosaccharides and subsequently into monosaccharides. The anaerobic environment of the distal colon, due to the removal of water and the abundance of substrate, is the appropriate situation for fermentation. Saccharolytic bacteria, such as *Bacteroidetes*, ferment the processed monosaccharides within the glycolysis and pentose‐phosphate pathways, to produce SCFAs in addition to the gases CO_2_ and H_2_.^23^, ^24^ From the main short‐chain fatty acids, acetate, propionate, and butyrate are present in an estimated ratio of 60:20:20 in the large intestine. Butyrate is produced by beneficial colonic bacteria and acts as the main source of energy for the colonocytes. Butyrate is beneficial to its metabolic effects on thermogenesis and inducing fibroblast growth factor 21 transcription to increase the oxidation of fatty acid and production of ketone bodies.^25^ Furthermore, butyrate has the capacity to improve the growth of *lactobacilli* and *bifidobacteria* in the colon.^26^ Patients with chronic hepatitis have shown a decrease in polyunsaturated lipids by disrupting the integrity of the intestinal barrier in the alteration of the gut microbiota composition; however, the total lipid has been increased.^14^ In addition, CHB patients have significantly lower levels of fecal bile acids with positive association with *F. prausnitzii*,* bifidobacteria* and lactic acid‐producing bacteria.^27^, ^28^ It has also been reported that intestinal dysbiosis in CHB patients was closely associated with the accumulation of serum metabolites, including aromatic amino acids phenylalanine and tyrosine that play a pathogenic role in liver disease.^14^

## 5 BACTERIAL LPS AND STIMULATION OF IMMUNE RESPONSES IN LIVER INJURY

Overproduction of lipopolysaccharide (LPS) as an endotoxin from the outer cell membrane of gram‐negative bacteria has been reported in acute or chronic hepatitis B patients.^29^, ^30^, ^31^ LPS stimulate inflammation in Toll‐like receptor 4 (TLR4) signaling pathway by ligation to the LPS‐binding protein (LBP), CD14, and the MD2.^32^ TLRs are responsible for recognizing specific pathogen‐associated molecular patterns (PAMP) to activate innate immune responses by the expression of proinflammatory cytokines.^33^ Upon the TLR4 ligation, both MyD88‐dependent transduction pathways and MyD88 induce an inflammatory response.^33^ In HBV transgenic mice, TLR activation induces intrahepatic IFN‐a/b production to suppress viral replication.^34^ However, in the tolerant immune phase of chronic hepatitis B, HBeAg suppresses TLR signaling result in inhibition of tumor necrosis factor alpha (TNF alpha) expression. HBeAg through its precore‐specific sequence binds to the Toll/IL‐1 receptor (TIR) motif to stop TLR signaling that induced by TLR2 or TLR4 ligands.^34^, ^35^ It is known that HSC and Kupffer cells express TLR4 which makes them sensitive to LPS.^15^ TLR4 signaling is a significant mediator of hepatic fibrosis in quiescent HSC by expression of proinflammatory factor‐kappa B (NF‐kB) factor in downstream of TLR4 activation.^36^

## 6 *E. COLI* COLONIZATION AND MECHANISM FOR DEVELOPMENT OF HCC

Patients in the end stage of liver disease show higher growth of *E. coli* and increase level of LPS ligand in circulation.^6^, ^15^, ^37^ The *E. coli* is a predominantly aerobic organism in the adult intestine, estimated at 10^7^‐10^8^ cfu (colony‐forming unit) in the colon and a minor number within the terminal ileum. *E. coli* dominates during the first year of life and becomes the main facultative anaerobic bacterium within the mature intestinal flora.^38^ This bacterium also contributes to the generation of an anaerobic environment that favors the further settlement of *Bacteroides*,* Bifidobacterium,* and *Clostridium* species. It also seems to be a harmless commensal that uses its host for a continuous source of nutritive, existing, and survivor. However, *E. coli* with a notable phenotypic variety of intestinal and extra intestinal pathogenic strains are associated with over 2 million human deaths per year.^39^, ^40^ The genetic structures of commensal *E. coli* are shaped by the various host and environmental characteristics that determine bacterial adaptation.^41^ *E. coli* strains can be classified into four main phylogenetic groups A, B1, B2 and D which differ in some features such as genome size and plasticity, virulence, and antibiotic resistance profiles.^42^, ^43^ These features are responsible in intestinal homeostasis in adulthood, as demonstrated by the transition from the phylogroups A to B2 in recent decades.^41^, ^43^, ^44^ In natural transmission of *E. coli* in rat from mothers to children, both genotoxic and nongenotoxic strains of B2 phylogroups were transferred to offspring and colonized the intestine of subsequent generations. Colibactin is a secondary metabolite that is synthesized by biosynthetic machinery complexes called Cytolethal Distending Toxins (CDTs) in B2 phylogroups. This toxin is like DNase, in structure and activity, which breaks the double‐strand DNA and induce cellular apoptosis.^41^, ^45^, ^46^ Colonization by colibactin‐producing B2 phylogroups at the early life of animal experiments associated with the reduction in regulatory T‐cell populations which is a susceptibility factor for the development of immune‐mediated diseases and the predisposition of cancer.^47^, ^48^, ^49^ The potential effect of intestinal *E. coli* on colorectal cancer development is extensively evaluated in animal and human models.^50^ *E. coli* LPS in conjunction with TLR4 triggers a powerful cytokine cascade that results in septic shock and death.^51^ Recent evidence has firmly confirmed the dynamic effect of intestinal dysbiosis on activation of TLR4 pathway in advanced liver disease. However, a number of tumor suppressors may elucidate the mechanism between the development of HCC and colonization of intestinal *E. coli*. As indicated above, colonic microbiota of patients with cirrhosis has revealed a growth of *Enterobacteriaceae* and *Enterococcus* but a decrease in *Bifidobacterium* species leading to endotoxemia with portal hypertension and damage to hepatocytes.^17^, ^19^ LPS derived from the microbiota binds to TLR4 and CD14, as a coreceptor, to activate the intracellular signaling pathways in upregulation of antiviral components.^30^, ^31^, ^33^ Kupffer cells and sinusoidal endothelial cells (LSEC) of nonparenchymal hepatic cells of mice in TLR4 stimulation, inhibit replication of HBV independently of MyD88, so TLR agonists may be useful for the treatment of chronic HBV infection.^52^ However, TNF alpha that is produced downstream of TLR4 signaling by activating the NF‐kB, linked to the hepatic inflammation and liver fibrosis.^32^, ^33^, ^53^ It has been demonstrated that the kinase activity of STK4 (serine/threonine kinase 4) damp down the production of proinflammatory cytokine that are activated in TLR pathways.^54^ The STK4 protein controls the TLR pathway by inhibiting the expression of NF‐kB. In mouse models with HCC that are infected with *E. coli,* the expression of STK4 in macrophages is reduced; however, IL‐6 and TNF alpha concentrations were inversely elevated.^54^ The higher expression of proinflammatory cytokines and the induction of hepatic fibrosis in the presence of LPS could be explained by the negative effect of *E. coli* on the lower expression of the STK4 tumor suppressor protein in patients with HBV.

## 7 CONCLUSIONS

The recent data summarized in this review display a weighted impact of intestinal microbiota and intestinal homeostasis on the progression of liver disease. Alterations in intestinal microbiota with excessive growth of *E. coli* with increased levels of bacterial LPS in the blood, involved in the development of HCC. However, future studies on the genetic profiles of *E. coli* in the gastrointestinal tract and its influence on the severity of liver disease in patients with CHB are recommended.

## CONFLICT OF INTEREST

None declared.
